# Effects of combined high pressure and enzymatic treatments on physicochemical and antioxidant properties of peanut proteins

**DOI:** 10.1002/fsn3.976

**Published:** 2019-03-05

**Authors:** Xin‐hong Dong, Jing Li, Guo‐xiang Jiang, Hai‐yun Li, Mou‐ming Zhao, Yue‐ming Jiang

**Affiliations:** ^1^ Department of Materials and Chemistry Engineering Guilin University of Technology Guilin China; ^2^ Key Laboratory of Plant Resource Conservation and Sustainable Utilization, Guangdong Provincial Key Laboratory of Applied Botany, South China Botanical Garden Chinese Academy of Sciences Guangzhou China; ^3^ College of Light Industry and Food Science South China University of Technology Guangzhou China

**Keywords:** antioxidant activity, enzymatic hydrolysis, high pressure, peanut protein, structure

## Abstract

Isolated peanut protein (PPI) dispersions were pretreated by high pressure at 100, 300, and 500 MPa prior to enzymatic hydrolysis with alkaline protease (Alcalase). The degree of hydrolysis (DH) was determined by the pH‐stat method, the hydrolysates profiles were analyzed by high‐performance liquid chromatography (HPLC), the molecular weight distribution (MWD) was analyzed by gel filtration chromatogram (GFC), and content of SH/S‐S and antioxidant activity of hydrolysates were evaluated. Results showed that HP pretreatment improved effectively the enzymatic hydrolysis of PPI, with an effective sequence of 300 > 100 > 500 MPa. However, no significant differences were observed in the peak pattern of HPLC profiles, but the peak times were earlier in HPLC profiles of the HP‐treated protein. GFC analysis showed that more peptide fractions with low molecular weight appeared in the hydrolysates of the HP‐treated PPI with increasing pressure. Moreover, the level of free SH of hydrolysates of the HP‐treated PPI was relatively higher than non‐HP‐treated PPI. The hydrolysates of the HP‐treated PPI exerted higher antioxidant activity (reducing power and DPPH radical scavenging) than the hydrolysates of non‐HP‐treated PPI. The results indicated that high pressure treatment affected the enzymatic hydrolysis of peanut protein and some protein structure properties and improved antioxidant activity of PPI hydrolysates.

## 1 INTRODUCTION

The industrial process of high pressure (HP) is a potential processing technique and used as an alternative to heat treatment in food industry (Chawla, Patil, & Singh, 2011). Many reports confirmed that HP treatment could improve some functional properties and then was used as a safe and effective modification method of proteins (Chicón, Belloque, Recio, & López‐Fandiño, 2006; Knudsen, Otte, Olsen, & Skibsted, 2002; Wang et al., 2008). Recent research confirmed that high pressure had an active impact on protein hydrolysis by increasing the proteolysis degree (Ambrosi, Polenta, Gonzalez, Ferrari, & Maresca, 2016; Peñas, Préstamo, Baezac, Martínez‐Molero, & Gomez, 2006; Zhao, Huo, Qian, Ren, & Lu, 2017). Peñas, Préstamo, and Gomez (2004) found that high pressure treatment of 100 MPa contributed to the hydrolysis of soy whey protein treated by different proteases. Belloque, Chicón, and López‐Fandiño (2007) reported that HP treatment at 200 MPa changed the structure, elasticity, and flexibility of proteins, which was related to the protease specificity and hydrophobic groups or aromatic groups embedded in the center of the original protein. Researchers pointed out that the original structure of β‐Lg in the HP‐treated protein disappeared immediately and the HP‐treated protein produced a large number of intermediate peptides, which would be hydrolyzed further by protease. In contrast, the original structure of protein without HP treatment was difficult to be hydrolyzed by protease (Belloque et al., 2007; Chicón, Belloque, Alonso, & López‐Fandiño, 2008).

Peanut is a major agricultural crop and is widely used for oil extraction. The by‐products of peanut after oil extraction comprise approximately 47%–55% of proteins which contain a significant amount of the essential amino acids and other valuable ingredients (Wu, Wang, Ma, & Ren, 2009; Yu, Ahmedna, & Goktepe, 2007). Thus, peanut by‐products can be utilized further in terms of their functional properties in other food products (Radha, Ramesh Kumar, & Prakash, 2007; Wu et al., 2009). As peanut oil is extracted at high temperature, the extraction results in various degree denaturations on protein and thus affects the physiochemical, functional properties, and some bioactivities (Aminigo & Ogundipe, 2003; Govindaraju & Srinivas, 2006; Mouécoucou, Villaume, Sanche, & Méjean, 2004). The objective of this work was to determine synergistic effects of high pressure and enzymatic hydrolysis on the structural and antioxidant properties of peanut protein. According to some characteristic change such as the hydrolysis degree of protein, profiles of high‐performance liquid chromatography (HPLC), and gel filtration chromatography (GFC), SH/S‐S content and antioxidant activity of hydrolysates, we understand the effects of two modification methods on the structural characteristic, functional properties, and biological activity of peanut proteins to utilize better peanut by‐products.

## 2 MATERIALS AND METHODS

### 2.1 Materials

Defatted peanut dregs as a by‐product from oil extraction of peanut seed were kindly supplied by Shandong Luhua Group Company (Shandong, China). The peanut dreg was pulverized in a mill (DFT‐50, Lingda Mechanics Co., Zhejiang, China), then passed through a 120‐mesh sieve, and finally, the peanut flour was obtained.

### 2.2 Preparation of peanut protein isolates (PPI)

Peanut protein isolates was prepared from the defatted peanut flour using the method described by Dong et al. (2011). The ratio of defatted peanut flour to distilled water was 1:8, and then, the pH of dispersion was adjusted to pH 8.5, and the dispersion was stirred for 1 hr at 60°C using a magnetic stirrer, then centrifuged at 3,000 *g* and 5°C for 10 min. The supernatant phase was collected again, then adjusted to pH 4.5 and centrifuged at 3,000 *g* and 5°C for 10 min. The precipitate was collected and redispersed in deionized water again. The solution was adjusted to pH 7.0 and then freeze‐dried to produce PPI for the next experiments.

### 2.3 High pressure processing of PPI

High pressure treatment was performed by the method of Tang and Ma (2009). Samples of PPI solutions were vacuumed in a polyethylene bag. PPI solutions at 5 mg/100 ml were treated by HP treatment at 100, 300, or 500 MPa for 20 min. The target pressure reached at a rate of about 250 MPa/min and released at a rate of about 300 MPa/min. The temperature of oil as transmitting medium was controlled at 25°C during the pressure processing. The PPI solutions after HP treatment were freeze‐dried for following enzymatic hydrolysis.

### 2.4 Enzymatic hydrolysis of PPI

The HP‐treated and non‐HP‐treated PPI dispersions (5%, w/v) were adjusted to pH 8.0 with 1 M NaOH, and then, Alcalase (135.94 µl/g) was added into these dispersions. The mixture of protein and enzyme was incubated at 53°C to start the enzymatic hydrolysis reaction. In this study, pH 8.0 was maintained by the addition of 1 M NaOH. The total hydrolysis time took 480 min, and these samples were withdrawn after 30, 60, 90, 120, 240, 360, and 480 min of hydrolysis, respectively. The hydrolysates were used for hydrolysis degree analysis. The degree of hydrolysis was determined by the pH‐stat method, as previously described by Dong et al. (2011).

### 2.5 High‐performance liquid chromatography (HPLC) analysis

The hydrolysates profiles were analyzed by HPLC as described by Izquierdo, Peñas, Baeza, and Gomez (2008). PPI hydrolysates (5 mg/ml) were dissolved in solvent A and then filtrated with a 0.45 mm polyether sulfone membrane prior to loading onto the column (20 ml injection loops). Operating conditions of HPLC (Shimadzu LC‐20 AT, Shimadzu Corporation, Japan; Vydac, 218 TP, 250 × 4.6 mm (C_18 _column); 5 µm particle size, Sigma‐Aldrich, St. Louis, MO, USA) were listed as follows: 25 (column temperature); flow rate: 0.7 ml/min, solvent A: 0.1% (v/v) trifluoroacetic acid (TFA) (sequential grade, Sigma, St. Louis, MO, USA) in deionized water; and solvent B: 0.08% (v/v) trifluoroacetic acid in acetonitrile‐deionized water (8:2). Elution procedure was performed by applying 5% B for 5 min, a linear gradient of 5%–75% B for 40 min, 75%–100% B for 2 min, 100%–5% B for 2 min, and then maintaining 5% B for 5 min. The absorbance was recorded at 220 nm.

### 2.6 Gel filtration chromatography analysis

The molecular weight distribution characteristic of hydrolysates was analyzed according to the method of Liu, Zhu, and Zhao (2008). Sephadex G25 gel column (1.0 × 80 cm) was used for the determination of molecular weight distribution. Sample (2.0 ml) at a concentration of 10 mg/ml was filtrated on a microporous membrane prior to loading onto the column. The samples were eluted with deionized water at a flow rate of 3 ml/10 min. The detection was carried out at the wavelength of 220 nm.

### 2.7 Determinations of free sulfhydryl (SH) and disulfide bond (SS) contents

The SH and S‐S groups contents were determined by the method of Cui, Zhou, Zhao, and Yang (2009). For determining free SH group level, 0.5 ml of peanut protein hydrolysates was mixed with 2.5 ml of Tris‐Gly‐8 M Urea and 0.02 ml of 4 mg/ml 5, 5′‐dithiobis‐2, 2′‐nitrobenzoic acid (DTNB). After 30 min of incubation at 25°C, the absorbance (A_412_) was recorded at 412 nm. The free SH group level was calculated by the following equation: free SH group level (µmol/g) = 73.53A_412_ × D/C, where D is the dilution coefficient, D = (0.5 + 2.5 + 0.02)/0.5 = 6.04, and C (mg/ml) is the protein concentration in tested sample.

For determination of the S‐S group level, the protein hydrolysates (0.2 ml) were mixed with 1.0 ml of 10 M Tris‐Gly‐10 M Urea and 0.02 ml of β‐mercaptoethanol. After incubation of 1 hr at 25°C, 10 ml of 12% (w/v) trichloroacetic acid was added and then stranded for 1 hr. Then, the sample was subjected to centrifugation at 3,000 *g* for 10 min. The residues were dissolved in 3 ml of Tris‐Gly‐8 M Urea and 0.03 ml of DTNB. After incubation of 30 min at 25°C, the absorbance at 412 nm was recorded. The S‐S group level was calculated by the following equation: S‐S group level (µmol/g) = 1/2 (total SH group level−SH group level) = 1/2 (73.53A_412_ × D/C−SH group level). Determination of total SH group level was same to the above free SH group level, where D = (3 + 0.03)/0.2 = 15.15, and C (mg/ml) is the protein concentration in tested sample.

### 2.8 Antioxidant activity of PPI hydrolysates

#### 2.8.1 Determination of reducing power

The reducing power of PPI hydrolysates was measured by the method of Dong et al. (2011). One milliliter of samples (5.0 mg/ml) was added into 2.5 ml of phosphate buffer (2.5 M, pH 7.0) and 2.5 ml of potassium ferricyanide (1%, w/v). The mixture was incubated for 20 min at 50°C, then 2.5 ml of 10% (w/v) trichloroacetic acid (TCA) was added, and finally, the reaction mixture was centrifuged for 15 min at 3,000 *g*. A total volume of 2.5 ml from the supernatant after centrifugation was collected and, then, mixed with 2.5 ml distilled water and 0.5 ml of 0.1% (w/v) ferric chloride. After incubation of 10 min at 25°C, the absorbance was recorded at 700 nm using a spectrophotometer. High absorbance of the reaction mixture indicated strong reducing power.

#### 2.8.2 Determination of DPPH radical scavenging activity

1,1‐Diphenyl‐2‐picrylhydrazyl (DPPH) radical scavenging activity of PPI hydrolysates was determined by the method of Cumby, Zhong, Naczk, and Shahidi (2008) with slight modifications. Sample solution (0.1 ml, 5 mg/ml) was mixed with 2.0 ml of 50 μM ethanolic DPPH solution and 0.9 ml distilled water. The mixture was allowed to stand for 30 min at 25°C. The absorbance was recorded at 517 nm using a spectrophotometer. The scavenging activity of DPPH by PPI hydrolysates was calculated as follow: DPPH scavenging activity (%) = 100 × $\frac{{\text{Abs}}_{\text{control}}-\left({\text{Abs}}_{\text{sample}}-{\text{Abs}}_{\text{blank}}\right)}{{\text{Abs}}_{\text{control}}}$, where the Abs_control _is the absorbance of DPPH without any hydrolysate and the Abs_blank_ represents the absorbance of the hydrolysates without DPPH while the Abs_sample_ is the absorbance of the hydrolysates with DPPH.

### 2.9 Statistical analysis

Data were expressed as means ± standard deviations (*SD*) of three replicated determinations. Analysis of variance (ANOVA) was applied to determining significant difference at *p* < 0.05 using SPSS 13.0 software (SPSS Inc., Chicago, IL, USA).

## 3 RESULTS AND DISCUSSION

### 3.1 Effect of high pressure treatment on enzymatic hydrolysis of PPI

**Figure 1:**
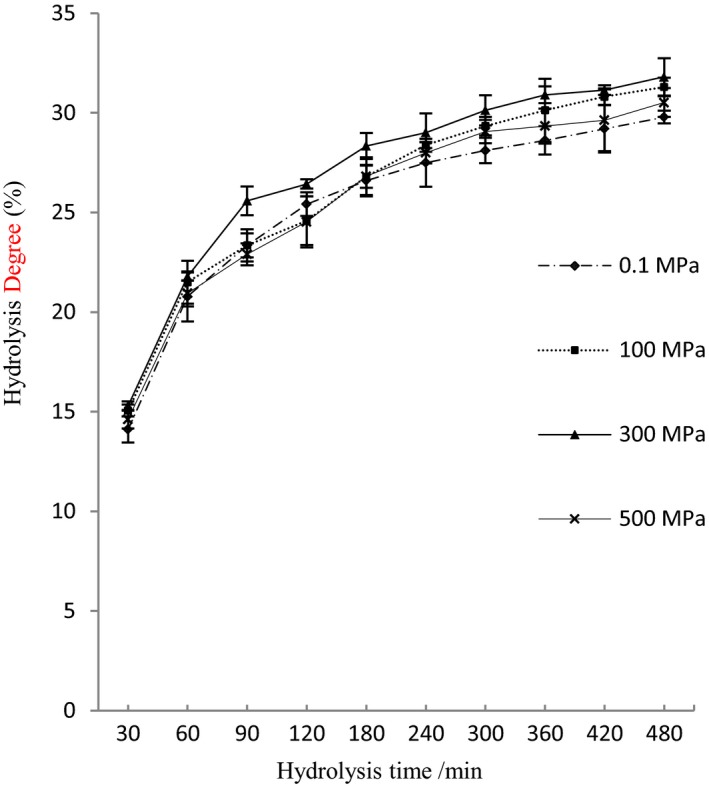
Effects of high pressure treatment on enzymatic hydrolysis of peanut protein

The time course of hydrolysis of the HP‐treated and non‐HP‐treated PPI is shown in Figure 1. The hydrolysis degree (DH) of the HP‐treated and non‐HP‐treated PPI presented a sharp rise within the first 60 min of hydrolysis. The DH of HP‐treated PPI was near or lower than that of non‐HP‐treated PPI within the first 180 min except the treatment at 300 MPa, which confirmed that peanut protein after high pressure treatment was not easy to be degraded efficiently by Alcalase. However, the DH of the HP‐treated PPI increased gradually with extending hydrolysis time and became higher than non‐HP‐treated PPI after 180 min of hydrolysis, which may be related to high pressure causing aggregation of some structures of proteins. Once the aggregations were damaged by the initial hydrolysis, the HP‐treated PPI was hydrolyzed more quickly than non‐HP‐treated PPI (Zhang, Olsen, Grossi, & Otte, 2013).

High pressure treatments enhanced the hydrolysis degree of peanut proteins compared to non‐HP‐treated proteins, with an effect sequence on the hydrolysis of 300 > 100 > 500 MPa. The DH of proteins treated by 300 MPa (after hydrolysis of 60 min) and 100 MPa (after hydrolysis of 180 min) was significantly higher (*p* < 0.05) than non‐HP‐treated proteins, but effect from 500 MPa treatment was not significant. It is presumed that the higher pressure (300 MPa) would result in the extension of protein structure, exposing more cleavage sites and increasing the susceptibility of PPI to proteolytic enzymes such as Alcalase (Peñas, Préstamo, Baezac et al., 2006). The DH under the 500 MPa treatment condition was lower than 100 and 300 MPa because some protein groups might gradually aggregate with increasing pressure so that the protein structure can become closer and more difficult to be hydrolyzed (Peñas et al., 2004).

### 3.2 High‐performance liquid chromatography (HPLC) analysis

**Figure 2:**
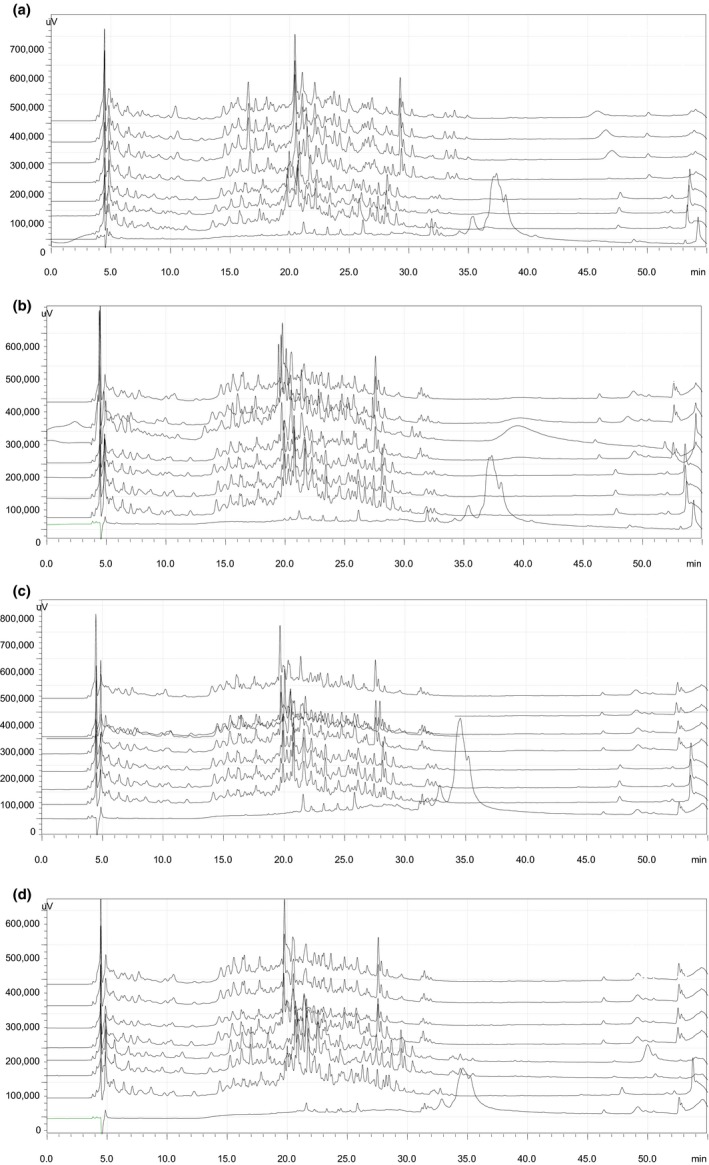
HPLC profiles of PPI hydrolysates under different pressure levels at 0.1, 100, 300, and 500 MPa during hydrolysis (0, 30, 60, 90, 120, 240, 360, and 480 min from the bottom up). (a) 0.1 MPa (control); (b) 100 MPa; (c) 300 MPa; (d) 500 MPa

As shown in Figure 2, there are similar peptide profiles between the hydrolysates of HP‐treated and non‐HP‐treated proteins, but different peak amounts and peak areas exist in real time, which is in accord with the results of Peñas, Préstamo, Polo, and Gomez (2006) and Zhang et al. (2013). They indicated that in general, the same peptide bonds were susceptibility to Alcalase irrespective of pretreatment. Similar phenomena were found also by Knudsen et al. (2002) who reported β‐lactoglobulin A was hydrolyzed by trypsin, chymotrypsin, and *B*. *licheniformis* protease after high pressure treatment (150, 300, and 450 MPa) and the hydrolysates from HP‐treated and non‐HP‐treated proteins had the similar hydrolysis rate and HPLC profile. However, a slightly different peptide profile maybe suggests that some protein structures were less accessible to Alcalase after high pressure treatment (Zhang et al., 2013). An interesting fact was observed that there is visible difference on the elution profile of hydrolysates before and after hydrolysis of 90 min. For non‐HP‐treated proteins, more elution peaks and wider elution zone occurred after the 90 min hydrolysis than the first 90 min, while for HP‐treated proteins, more elution peaks and wider elution zone appeared within the first 90 min than after 90 min. Maybe it is because the peptide fractions from the HP‐treated proteins became less due to the higher hydrolysis degree.

As for the proteins without hydrolysis, there are only one big peak and few small peaks in the HPLC profile, but the peak time of the HP‐treated proteins (300 and 500 MPa, 33.6–36.5 min) was earlier than non‐HP‐treated proteins (0.1 and 100 MPa, 36.1–40 min) with increasing pressure, which indicated that high pressure treatments affected greatly the structure of intact protein and, thus, changed the elution time.  However, HP‐treated and non‐HP‐treated proteins were hydrolyzed rapidly by Alcalase at the early stage of hydrolysis, with a large number of released peptides (Figure 1). The peak time of abundant of peptides mostly occurred at 14–30 min, and most peptide fractions of hydrolysates after the hydrolysis of 30 min partially converted into some hydrophilic peptides with a shorter retention time and hydrophobic peptides with a longer retention time during hydrolysis.

### 3.3 Molecular weight distribution of hydrolysates

**Figure 3:**
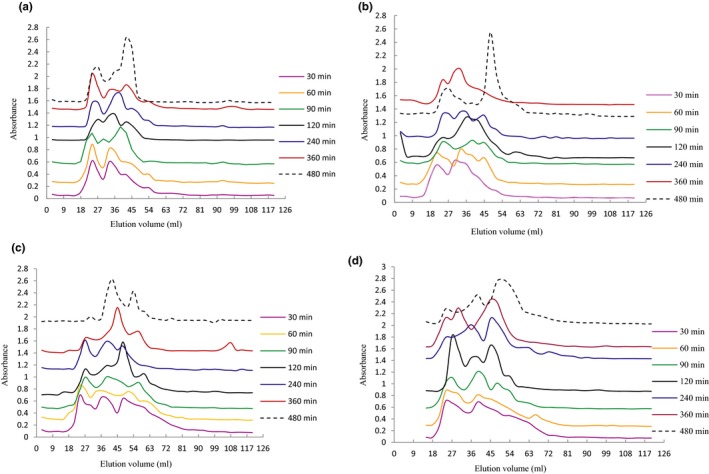
Molecular weight distribution of hydrolysates of PPI under different pressure levels at 0.1, 100, 300, and 500 MPa during hydrolysis (0, 30, 60, 90, 120, 240, 360, and 480 min). (a) 0.1 MPa (control); (b) 100 MPa; (c) 300 MPa; (d) 500 MPa

There was an obvious difference on the molecular weight distribution of hydrolysates between HP‐treated and non‐HP‐treated proteins. The hydrolysates of non‐HP‐treated and 100 MPa‐treated proteins had a similar molecular weight distribution pattern (Figure 3a,b). There were some small peaks in the hydrolysates from different hydrolysis time while a big peak at the late elution time (low molecular peptides) was observed in the hydrolysates after the hydrolysis of 480 min, which meant that intact proteins were hydrolyzed gradually into many low molecular peptides.

The distribution characteristic of Figure 3c,d showed more peaks and wider distribution zone, which indicated that there were more complicated peptide composition and more small peptides in the hydrolysates of proteins treated by high pressure. Knudsen et al. (2002) also found that high pressure treatment at 150 MPa had little impact on the molecular distribution of β‐lactoglobulin A, while high pressure from 300 to 450 Mpa had an obvious influence on the molecular distribution, which caused the formation of more monomer structure and oligomer. Moreover, the big peaks existed formerly at the late elution time (Figure 3a,b) disappeared in the Figure 3c,d, which indicated that these low molecular peptides were hydrolyzed further, with a higher hydrolysis degree, which was in accordance with the results of Figure 1.

### 3.4 Contents of free sulfhydryl (SH) and disulfide bond (S‐S)

**Figure 4:**
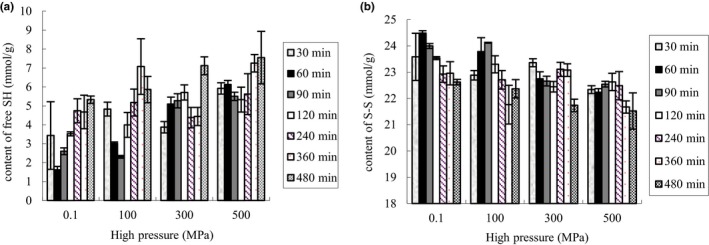
Comparison of hydrolysates of PPI at different pressure levels during hydrolysis time. (a) Content of free sulfhydryl group; (b) content of S‐S group

The analyses of thiol groups and disulfides provide important information on the conformational structure and stability of the proteins (Ambrosi et al., 2016). Previous reports indicated that high pressure treatment caused the change in the structure of proteins to some degrees, which depends largely on the experimental condition (Huppertz, Smiddy, Upadhyay, & Kelly, 2006). Figure 4 reflects the total SH group, free SH group, and S‐S of hydrolysates of PPI after different high pressure treatments. Total SH group level of non‐HP‐treated proteins was 47.94 ± 7.83 µmol/g while the proteins treated by 100, 300, and 500 MPa were 49.04 ± 0.52, 51.47 ± 4.02, and 51.25 ± 2.56 µmol/g, respectively. However, high pressure treatments did not affect significantly the total SH group level.

Figure 4a shows the change in free SH content of PPI during enzymatic hydrolysis. The contents of the SH group of HP‐treated samples were higher than non‐HP‐treated samples. The SH group levels of all the samples were reduced firstly and increased subsequently with prolonging hydrolysis time, with a delayed time of the lowest SH group contents with increasing pressure. SH group contents of non‐HP‐treated and 100 MPa‐treated samples reduced at 60 min and increased from 120 min while they after 300 and 500 MPa treatments reduced at 240 and 120 min and increased from 480 and 360 min, respectively. Some reports concluded that high pressure treatment led to the reversible unfolding and refolding of the proteins after pressure was released. When pressure was released, unfolded protein molecules that did not interact with other proteins may refold to their native state, so decreased SH group contents were observed (Ambrosi et al., 2016; Belloque et al., 2007; Huppertz, Fox, & Kelly, 2004).

The change in S‐S content of PPI during the enzymatic hydrolysis is shown also in Figure 4b. By comparing the change in the SH and S‐S group level of PPI hydrolysates with and without HP treatment, the variation range of SH group was determined to be from 1.63 ± 0.18 to 7.55 ± 1.39 µmol/g while S‐S was determined to be from 21.5 2 ± 0.69 to 24.49 ± 0.09 µmol/g, which indicated that S‐S was the major structure of the peanut proteins and acted as the important factor to maintain dimensional structure and functional properties of PPI. Previous reports concluded that the formation and changes of monomer structure and oligomer in the PPI hydrolysates may be related to protein aggregation during high pressure treatment and small molecular peptides released by enzymatic hydrolysis could be mutual cross‐linking by the interaction of SH/S‐S and oxidation of SH group (Lai et al., 2010; Owusu‐Apenten, 2005; Van der Plancken, Van Loey, & Hendrickx, 2005).

### 3.5 Antioxidant activity of PPI hydrolysates

**Figure 5:**
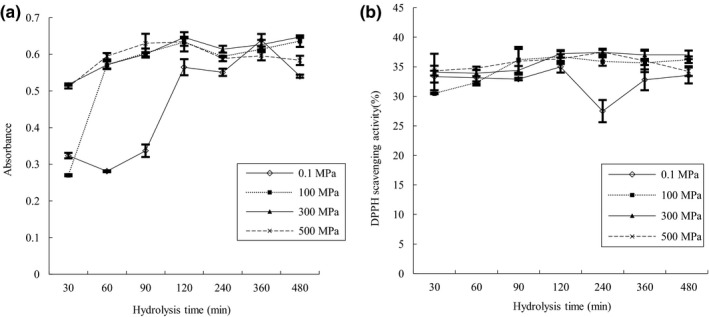
Effects of high pressure on antioxidant activity of hydrolysates. (a) Reducing power of hydrolysates; (b) DPPH free radical scavenging activity of hydrolysates

It can be seen from Figure 5a that the reducing power of all samples exhibited an increasing trend within the first 120 min of hydrolysis and the reducing power of HP‐treated samples was significantly (*p* < 0.05) higher than non‐HP‐treated samples. Higher reducing power showed that more antioxidant peptides were produced with increasing hydrolysis degree within the first 120 min. The result explained that enzymatic hydrolysis could result in released peptides from peanut proteins and an optimum degree of hydrolysis could lead to the PPI hydrolysates with the highest antioxidant activity (Zhang et al., 2013). With further hydrolysis, because the compositions and contents of these antioxidant peptides tended to be stable, the reducing power was no longer to be increased. Thus, the high reducing power of peanut protein hydrolysates was attributed to increasing availability of hydrogen ions (protons and electrons) due to peptide cleavages (Liu, Kong, Xiong, & Xia, 2010).

The Figure 5b shows the difference in DPPH free radical scavenging activity. The DPPH free radical scavenging activity of HP‐treated hydrolysates increased with hydrolysis time and hydrolysis degree, and tended to maintain a stable level. The DPPH free radical scavenging activity of the hydrolysate of non‐HP‐treated proteins increased gradually within the first 120 min of hydrolysis and then declined sharply after 240 min, followed by an increase. The DPPH free radical scavenging activity of HP‐treated hydrolyates was significantly higher than non‐HP‐treated hydrolysates, which suggested that high pressure combined with enzymatic hydrolysis can be an efficient way to increase DPPH free radical scavenging activity of PPI.

## 4 CONCLUSION

High pressure treatment of PPI solution changed the biochemical properties of PPI and enhanced the susceptibility to enzymatic hydrolysis. It was found that application of 300 MPa pretreatment was more effective for the enzymatic hydrolysis and release of peptides as compared to 100 or 500 MPa treatment. Combined high pressure and enzymatic hydrolysis treatments affected also the structural properties to different extents, such as peptides profile, molecular weight distribution, contents of SH group, and S‐S group. Simultaneously, high pressure treatment combined with enzymatic hydrolysis resulted in increased antioxidant activity of PPI hydrolysates. Results indicated that combined high pressure and enzymatic hydrolysis had the potential to induce the change in protein structure, which favored enzymatic hydrolysis to modify the protein functional properties. For elucidation of synergistic role of high pressure and enzymatic hydrolysis, the isolation, purification, and structural identification of antioxidant and other bioactive peptides need to be investigated further.

## CONFLICT OF INTEREST

The authors declare no conflict of interest.

## ETHICAL STATEMENT

This study does not involve any human or animal testing.

## References

[fsn3976-bib-0001] Ambrosi, V. , Polenta, G. , Gonzalez, C. , Ferrari, G. , & Maresca, P. (2016). High hydrostatic pressure assisted enzymatic hydrolysis of whey proteins. Innovative Food Science and Emerging Technologies, 38, 294–301. 10.1016/j.ifset.2016.05.009

[fsn3976-bib-0002] Aminigo, E. R. , & Ogundipe, H. O. (2003). Effect of heat treatment on functional characteristics of peanut (*Arachis hypogeae*) meal. Journal of Food Science and Technology, 40, 205–208.

[fsn3976-bib-0003] Belloque, J. , Chicón, R. , & López‐Fandiño, R. (2007). Unfolding and refolding of β‐lactoglobulin subjected to high hydrostatic pressure at different pH values and temperatures and its influence on proteolysis. Journal of Agricultural and Food Chemistry, 55, 5282–5288. 10.1021/jf070170w 17542606

[fsn3976-bib-0004] Chawla, R. , Patil, G. R. , & Singh, A. K. (2011). Hydrostatic pressure technology in dairy processing: A review. Journal of Food Science and Technology, 48, 260–268. 23572744 10.1007/s13197-010-0180-4 PMC3551163

[fsn3976-bib-0005] Chicón, R. , Belloque, J. , Alonso, E. , & López‐Fandiño, R. (2008). Immunoreactivity and digestibility of high‐pressure‐treated whey proteins. International Dairy Journal, 18, 367–376. 10.1016/j.idairyj.2007.11.010

[fsn3976-bib-0006] Chicón, R. , Belloque, J. , Recio, I. , & López‐Fandiño, R. (2006). Influence of high hydrostatic on the proteolysis of β‐lactoglobulin A by trypsin. Journal of Dairy Research, 73, 121–128. 16433971 10.1017/S0022029905001664

[fsn3976-bib-0007] Cui, C. , Zhou, X. S. , Zhao, M. M. , & Yang, B. (2009). Effect of thermal treatment on the enzymatic hydrolysis of chicken proteins. Innovative Food Science and Emerging Technologies, 10, 37–41. 10.1016/j.ifset.2008.09.003

[fsn3976-bib-0008] Cumby, N. , Zhong, Y. , Naczk, M. , & Shahidi, F. (2008). Antioxidant activity and water‐holding capacity of canola protein hydrolysates. Food Chemistry, 109, 144–148. 10.1016/j.foodchem.2007.12.039 26054275

[fsn3976-bib-0009] Dong, X. H. , Zhao, M. M. , Shi, J. , Yang, B. , Li, J. , Luo, D. H. , … Jiang, Y. M. (2011). Effects of combined high‐pressure homogenization and enzymatic treatment on extraction yield, hydrolysis and function properties of peanut proteins. Innovative Food Science and Emerging Technologies, 12, 478–483. 10.1016/j.ifset.2011.07.002

[fsn3976-bib-0010] Govindaraju, K. , & Srinivas, H. (2006). Studies on the effects of enzymatic hydrolysis on functional and physicochemical properties of arachin. Swiss Society of Food Science and Technology, 39, 54–62.

[fsn3976-bib-0011] Huppertz, T. , Fox, P. F. , & Kelly, A. L. (2004). High pressure‐induced denaturation of α‐lactalbumin and β‐lactoglobulin in bovine milk and whey: A possible mechanism. Journal of Dairy Research, 71, 489–495. 15605716 10.1017/s0022029904000500

[fsn3976-bib-0012] Huppertz, T. , Smiddy, M. A. , Upadhyay, V. , & Kelly, A. L. (2006). High pressure‐induced changes in bovine milk: A review. International Journal of Dairy Technology, 59, 58–66. 10.1111/j.1471-0307.2006.00246.x

[fsn3976-bib-0013] Izquierdo, F. J. , Peñas, E. , Baeza, M. L. , & Gomez, R. (2008). Effects of combined microwave and enzymatic treatments on the hydrolysis and immunoreactivity of dairy whey proteins. International Dairy Journal, 18, 918–922. 10.1016/j.idairyj.2008.01.005

[fsn3976-bib-0014] Knudsen, J. C. , Otte, J. , Olsen, K. , & Skibsted, L. H. (2002). Effect of high hydrostatic pressure on the conformation of β‐lactoglobulin A as assessed by proteolytic peptide profiling. International Dairy Journal, 12, 791–803.

[fsn3976-bib-0015] Lai, K. M. , Chuang, Y. S. , Chou, Y. C. , Hsu, Y. C. , Cheng, Y. C. , Shi, C. Y. , … Hsu, K. C. (2010). Changes in physicochemical properties of egg white and yolk proteins from duck shell eggs due to hydrostatic pressure treatment. Poultry Science, 89, 729–737. 10.3382/ps.2009-00244 20308405

[fsn3976-bib-0016] Liu, L. J. , Zhu, C. H. , & Zhao, Z. (2008). Analyzing molecular weight distribution of whey protein hydrolysates. Food and Bioproducts Processing, 86, 1417–6. 10.1016/j.fbp.2007.10.007

[fsn3976-bib-0017] Liu, Q. , Kong, B. H. , Xiong, Y. L. , & Xia, X. F. (2010). Antioxidant activity and functional properties of porcine plasma protein hydrolysate as influenced by the degree of hydrolysis. Food Chemistry, 118, 403–410. 10.1016/j.foodchem.2009.05.013

[fsn3976-bib-0018] Mouécoucou, J. , Villaume, C. , Sanche, C. , & Méjean, L. (2004). Effects of gum arabic, low methoxy pectin and xylan on in vitro digestibility of peanut protein. Food Research International, 37, 777–783. 10.1016/j.foodres.2004.04.002

[fsn3976-bib-0019] Owusu‐Apenten, R. (2005). Colorimetric analysis of protein sulfhydryl groups in milk: Applications and processing effects. Critical Reviews in Food Science and Nutrition, 45, 1417–23. 10.1080/10408690590900126 15730186

[fsn3976-bib-0020] Peñas, E. , Préstamo, G. , Baezac, M. L. , Martínez‐Molero, M. I. , & Gomez, R. (2006). Effects of combined high pressure and enzymatic treatments on the hydrolysis and immunoreactivity of dairy whey proteins. International Dairy Journal, 16, 831–839.

[fsn3976-bib-0021] Peñas, E. , Préstamo, G. , & Gomez, R. (2004). High pressure and the enzymatic hydrolysis of soybean whey proteins. Food Chemistry, 85, 641–648. 10.1016/j.foodchem.2003.07.025

[fsn3976-bib-0022] Peñas, E. , Préstamo, G. , Polo, F. , & Gomez, R. (2006). Enzymatic proteolysis, under high pressure of soybean whey: Analysis of peptides and the allergen Gly m 1 in the hydrolysates. Food Chemistry, 99, 569–573.

[fsn3976-bib-0023] Radha, C. , Ramesh Kumar, P. , & Prakash, V. (2007). Preparation and characterization of a protein hydrolysate from an oil seed flour mixture. Food Chemistry, 106, 1166–1174.

[fsn3976-bib-0024] Tang, C. H. , & Ma, C. Y. (2009). Effect of high pressure treatment on aggregation and structural properties of soy protein isolate. LWT ‐ Food Science and Technology, 42, 606–611. 10.1016/j.lwt.2008.07.012

[fsn3976-bib-0025] Van der Plancken, I. , Van Loey, A. , & Hendrickx, M. E. G. (2005). Changes in sulfhydryl content of egg white proteins due to heat and pressure treatment. Journal of Agricultural and Food Chemistry, 53, 5726–5733. 10.1021/jf050289+ 15998140

[fsn3976-bib-0026] Wang, X.‐S. , Tang, C.‐H. , Li, B.‐S. , Yang, X.‐Q. , Li, L. , & Ma, C.‐Y. (2008). Effects of high‐pressure treatment on some physicochemical and functional properties of soy protein isolates. Food Hydrocolloids, 22, 560–567. 10.1016/j.foodhyd.2007.01.027

[fsn3976-bib-0027] Wu, H. W. , Wang, Q. , Ma, T. Z. , & Ren, J. J. (2009). Comparative studies on the functional properties of various protein concentrate preparations of peanut protein. Food Research International, 42, 343–348. 10.1016/j.foodres.2008.12.006

[fsn3976-bib-0028] Yu, J. M. , Ahmedna, M. , & Goktepe, I. (2007). Peanut protein concentrate: Production and functional properties was affected by processing. Food Chemistry, 103, 121–129.

[fsn3976-bib-0029] Zhang, Y. H. , Olsen, K. , Grossi, A. , & Otte, J. (2013). Effect of pretreatment on enzymatic hydrolysis of bovine collagen and formation of ACE‐inhibitory peptides. Food Chemistry, 141, 2343–2354. 10.1016/j.foodchem.2013.05.058 23870967

[fsn3976-bib-0030] Zhao, R. J. , Huo, C. Y. , Qian, Y. , Ren, D. F. , & Lu, J. (2017). Ultra‐high‐pressure processing improves proteolysis and release of bioactive peptides with activation activities on alcohol metabolic enzymes in vitro from mushroom foot protein. Food Chemistry, 231, 25–32. 10.1016/j.foodchem.2017.03.058 28450004

